# Investigating Binge Eating Using Ecological Momentary Assessment: The Importance of an Appropriate Sampling Frequency

**DOI:** 10.3390/nu10010105

**Published:** 2018-01-19

**Authors:** Tobias D. Kockler, Philip S. Santangelo, Ulrich W. Ebner-Priemer

**Affiliations:** Mental mHealth Lab, Karlsruhe Institute of Technology, 76187 Karlsruhe, Germany; philip.santangelo@kit.edu (P.S.S.); ulrich.ebner-priemer@kit.edu (U.W.E.-P.)

**Keywords:** binge eating, ecological momentary assessment, sampling frequency

With great interest, we read the recently published review on emotion regulation in binge eating disorder (BED) by Dingemans et al. [1]. We fully agree with the authors that (a) in order to better understand binge eating, it is of major importance to delineate its affective consequences; and (b) ecological momentary assessment (EMA) is the gold standard to track these affective dynamics in patients’ everyday lives without retrospective distortions [2]. Whereas Dingemans et al. [1] are surprised that empirical evidence supporting the theoretical models is inconclusive, yet all models assume a reduction of negative affect after binge eating episodes, we can provide a coherent explication for the lack of evidence—namely the sampling frequency.

Most of the studies included in the meta-analysis reported by Dingemans and colleagues [2] tracked a highly dynamic process (affective consequences) using a low sampling frequency. However, a low sampling frequency is not able to capture rapid affective dynamics. In detail, the average sampling frequency in the meta-analysis was approximately 7.8 times per day [3], which corresponds to one assessment every two hours during the waking time. Our case example (see Figure 1) depicts the underlying velocity of affective dynamics.

We extracted a 1.5-h time segment from an EMA study, in which we tracked patients with bulimia nervosa (BN)—among other disorders—every 15 min (±1 min) over a period of 24 h during their waking time [4]. The extracted 1.5-h time segment covers seven separate assessments, and revealed impressive dynamics. In detail, at 4:56 p.m., the patient reported interest as her predominant momentary emotion, a positive emotional valence, and an urge to eat as well as an aversive tension at a medium level. Fifteen minutes later, the patient switched to an angry mood state and intense negative valence. At 5:26 p.m., she began with regular eating accompanied by the feeling of disgust, which then turned to loss-of-control binge eating at the next two assessment points until 5:58 p.m. While binge eating, the patient first felt disgust followed by anxiety. The urge to eat, aversive tension, and negative valence reached their climax in the middle of the eating episode. Whereas the urge to eat was already decreasing at the end of the eating episode, the aversive tension declined immediately afterwards. Interestingly, this decline of tension was accompanied by a strong feeling of joy from 6:12 p.m. to 6:26 p.m. We would have missed all these dynamics using a lower sampling frequency, such as one assessment every two hours or assessments just before and after binge eating episodes.

What applies to binge eating episodes also holds true for binge-purge episodes, which we provide an illustrative example of in Figure 2. During a 1.5-h binge-purge episode of another patient with BN, we see remarkable dynamics in “urge to eat” and “urge to vomit” accompanied by a transition from sadness to disgust at the beginning of the purging behavior. This is in line with Dingemans et al. [1], who conclude that binge eating episodes and binge-purge cycles may be different processes with specific affective dynamics.

In summary, the sampling frequency must fit the dynamics of the process of interest in order to allow for valid conclusions [5]. Future EMA studies testing the theoretical models of BED may benefit from an appropriate sampling frequency.

## Figures and Tables

**Figure 1:**
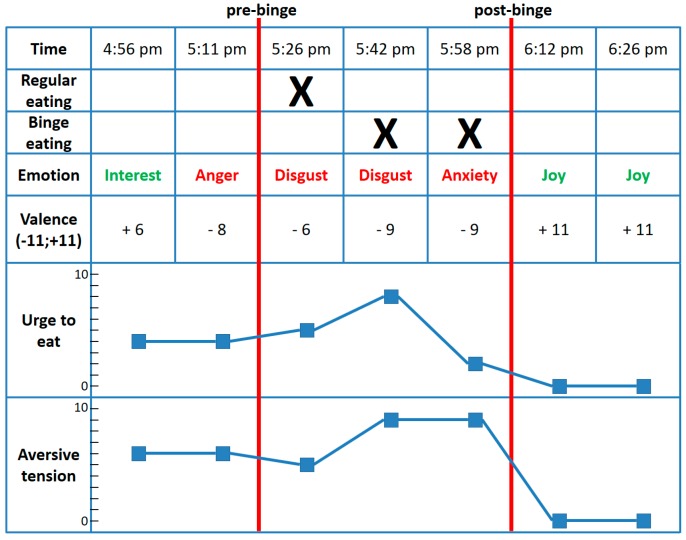
Affective dynamics and eating behavior of a single patient with bulimia nervosa (BN) before, during, and after a binge eating episode. Valence constitutes the intensity of emotions rated on an 11-point Likert scale from 0–11, whereas the intensity rating was multiplied by −1 in the case of negative emotions; therefore, valence scores range from −11 to +11. Urge to eat and aversive tension were assessed on an 11-point Likert scale from 0–10, with higher values indicating a stronger urge to eat and stronger aversive tension, respectively.

**Figure 2:**
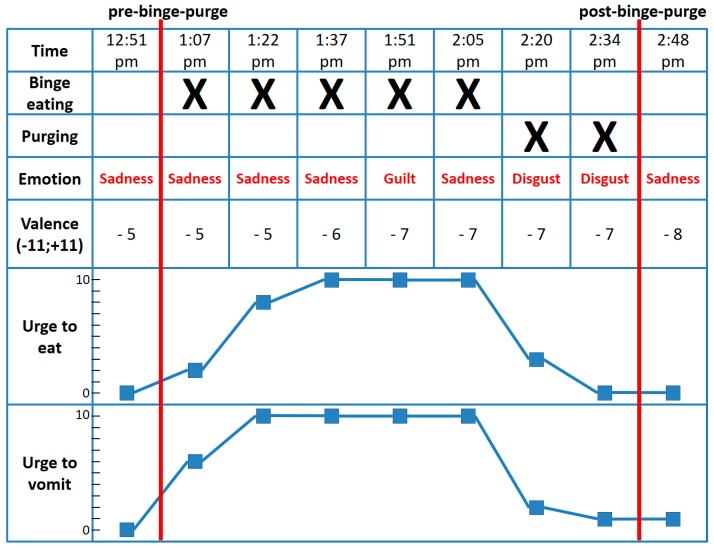
Affective dynamics, eating behavior, and purging behavior of a single patient with bulimia nervosa (BN) before, during, and after a binge-purge episode. Valence constitutes the intensity of emotions rated on a 11-point Likert scale from 0–11, whereas the intensity rating was multiplied by −1 in the case of negative emotions; therefore, valence scores range from −11 to +11. Urge to eat and urge to vomit were assessed on a 11-point Likert scale from 0–10, with higher values indicating a stronger urge.
